# Adaptive Aggregation Routing to Reduce Delay for Multi-Layer Wireless Sensor Networks

**DOI:** 10.3390/s18041216

**Published:** 2018-04-16

**Authors:** Xujing Li, Anfeng Liu, Mande Xie, Neal N. Xiong, Zhiwen Zeng, Zhiping Cai

**Affiliations:** 1 School of Information Science and Engineering, Central South University, Changsha 410083, China; xujingli@csu.edu.cn (X.L.); afengliu@mail.csu.edu.cn (A.L.); zengzhiwen@mail.csu.edu.cn (Z.Z.); 2 The State Key Laboratory of Industrial Control Technology, Zhejiang University, Hangzhou 310027, China; 3 School of Computer Science and Information Engineering, Zhejiang Gongshang University, Hangzhou 310018, China; 4 Department of Mathematics and Computer Science, Northeastern State University, Tahlequah, OK 74464, USA; xiongnaixue@gmail.com; 5 Department of Network Engineering, School of Computer, National University of Defense Technology, Changsha 410073, China; zpcai@nudt.edu.cn

**Keywords:** wireless sensor networks, data aggregation, aggregation delay, aggregation ratio, energy efficiency

## Abstract

The quality of service (QoS) regarding delay, lifetime and reliability is the key to the application of wireless sensor networks (WSNs). Data aggregation is a method to effectively reduce the data transmission volume and improve the lifetime of a network. In the previous study, a common strategy required that data wait in the queue. When the length of the queue is greater than or equal to the predetermined aggregation threshold ($N_{t}$) or the waiting time is equal to the aggregation timer ($T_{t}$), data are forwarded at the expense of an increase in the delay. The primary contributions of the proposed Adaptive Aggregation Routing (AAR) scheme are the following: (a) the senders select the forwarding node dynamically according to the length of the data queue, which effectively reduces the delay. In the AAR scheme, the senders send data to the nodes with a long data queue. The advantages are that first, the nodes with a long data queue need a small amount of data to perform aggregation; therefore, the transmitted data can be fully utilized to make these nodes aggregate. Second, this scheme balances the aggregating and data sending load; thus, the lifetime increases. (b) An improved AAR scheme is proposed to improve the QoS. The aggregation deadline ($T_{t}$) and the aggregation threshold ($N_{t}$) are dynamically changed in the network. In WSNs, nodes far from the sink have residual energy because these nodes transmit less data than the other nodes. In the improved AAR scheme, the nodes far from the sink have a small value of $T_{t}$ and $N_{t}$ to reduce delay, and the nodes near the sink are set to a large value of $T_{t}$ and $N_{t}$ to reduce energy consumption. Thus, the end to end delay is reduced, a longer lifetime is achieved, and the residual energy is fully used. Simulation results demonstrate that compared with the previous scheme, the performance of the AAR scheme is improved. This scheme reduces the delay by 14.91%, improves the lifetime by 30.91%, and increases energy efficiency by 76.40%.

## 1. Introduction

In the past several decades, there have been a variety of developments in wireless sensor networks (WSNs) in both academia and industry [1,2,3,4,5]. A wireless sensor network is a set of distributed hardware-constrained wireless devices that monitor a targeted area [6,7,8,9,10,11]. Wireless sensor networks are widely used in a variety of applications such as the environmental monitoring and urban health monitoring [12,13,14], healthcare [2,15], vehicular communication networks [16], cyber-physical cloud systems [17], multi-channel cognitive radio networks [18,19], crowdsourcing networks [20,21,22], and social networks [23,24]. Because of the growing demand for wireless sensor applications in various aspects, quality of service (QoS) is an important issue in wireless sensor applications [25,26,27,28,29].

For the QoS of wireless sensor applications, energy efficiency [5,11,17,30,31] and delay [8,9,27,29] are two of the most significant indicators. Since wireless sensor nodes are battery-powered, their energy is limited. Thus, improving energy efficiency [14,15,23,28,32,33,34] is an important research area for WSNs. There are many studies regarding energy efficiency, which include optimization in a single layer and joint optimization in multiple layers. For instance, optimization methods in the MAC layer [2,11,26], network layer and application layer, and cross-layer optimization methods have been proposed. Methods that address the MAC layer include adjusting the transmission power of the nodes [2,11,26], selecting optimized MAC parameters [2,11,26], and the dynamic duty cycle [35]. The network routing optimization method in the network layer [7,32] and selecting the optimal size of data packets in the application layer [2] are effective methods, as well. Among these methods, data aggregation is an effective and widely studied method [7,29,36,37,38]. Since there is a correlation between sensing data, the amount of data packets after aggregation is less than the original amount when two or more data packets aggregate, thereby reducing the number of network data packets [36,37,38]. Receiving and sending data packets consumes the most energy in sensing nodes; therefore, reducing the number of data packets to be transmitted can reduce energy consumption. Therefore, improving the efficiency of data aggregation is an effective approach to improving network energy efficiency. An important principle for improving the data aggregation efficiency requires data packets to encounter as many other data packets possible during the process of data collection to increase the probability of data aggregation [29,36,38]. In many data aggregation methods, when a node receives data packets, it does not send them immediately but holds them for a period of time [29] to increase the number of data packets waiting in the aggregation queue. When the number of data packets in a node is the same or greater than the predetermined threshold or the aggregation timer (AT) expires, the data packets are aggregated and transmitted. However, this effect causes an increase in the delay. With a large AT value, data aggregation works well, the data aggregation energy consumption decreases, but the delay significantly increases. The relationship between the delay, the length of the data queue and the AT has been confirmed by researchers. The average aggregation delay linearly increases as the aggregation timer or the aggregation threshold increases [29].

Although researchers have confirmed the optimization relationship, improving the QoS of the wireless sensor network remains a challenging issue [2]. The current research in optimizing aggregation delay is confined to networks with a star topology [29]. No study has been proposed to improve the QoS for general planar WSNs. The previous conclusions may provide guidance for the optimization of general planar WSNs. Planar WSNs are more complicated than star WSNs. The planar wireless sensor network is abstracted as a tree network in many studies. In this kind of network, the root of the network is the sink [39,40]. Other nodes in the network receive data from their child nodes, except for leaf nodes. There may be multiple children under a parent node, and each node may have multiple parents [26,41]. There are diverse routing paths for a node to transmit its aggregation queues. It dynamically changes the structure of the network and delay is affected. These influences interfere with each other. Thus, proposing an optimization strategy for data aggregation in a planar network is very challenging.

In this paper, an adaptive aggregation routing (AAR) scheme is proposed to reduce the delay and improve the lifetime for multi-layer wireless sensor networks. The main innovations of this paper are as follows:

(1) An adaptive aggregation routing (AAR) scheme is proposed to reduce delay and improve the lifetime for multi-layer wireless sensor networks. The core of this scheme is the node assignment algorithm (NAAL) that we proposed. This algorithm addressed the assignment of data queues in two adjacent layers of the network. According to the state of the data queue in the nodes, this algorithm selects nodes with a long data queue in the upper layer and sets the priority according to the length of the data queue. These nodes are guaranteed to receive sufficient data to aggregate, while other nodes which have no data to send are put to sleep in this process to save energy. Therefore, the frequency of data aggregation increases and the total delay decreases. Simulation results illustrate that the AAR scheme reduces the delay by 14.91% and improves the lifetime by 30.91% compared to other common schemes.
(2) Based on the AAR scheme, an improved optimization method is proposed in this paper, which improves the QoS. The main idea of the improved ARR scheme is as follows: the data in the nodes far from the sink use more hops to be transmitted to the sink, and there is delay in every hop. Consequently, there is noticeable delay caused during the process of transmitting the data from the distant nodes to the sink. In WSNs, nodes far away from the sink transmit less data than the other nodes. Therefore, more energy remains in these nodes. In the improved AAR scheme, the nodes far away from the sink have a small aggregation deadline ($T_{t}$) value and queue length threshold ($N_{t}$) to improve the frequency of sending the aggregation queues. The nodes near the sink are set to a large $T_{t}$ value and $N_{t}$ value to reduce energy consumption. The total delay is reduced, high lifetime is achieved, and the residual energy is fully used. Based on the simulations, the improved AAR scheme increases the energy efficiency by 76.40%.
(3) To evaluate its effectiveness, we conducted extensive simulations in a variety of network environments. The results indicate that the scheme we proposed performs better than other common networks.

The rest of this paper is organized as follows: in Section 2, related works are reviewed. The system model and problem statement are described in Section 3. In Section 4, design details on AAR scheme are presented. Section 5 is experiment results and comparisons for AAR scheme. Finally, Section 6 provides the study’s conclusions.

## 2. Related Work

This section presents previous work related to optimizing energy efficiency and delay in wireless sensor networks (WSNs).

### 2.1. Research on Data Aggregation

Data aggregation is a major operation in WSNs [7,29,36,37,38]. Sensor nodes are deployed in a certain area, and the sensing data are correlated in time and space. As a result, nodes aggregate data to a smaller size. Different research methods have been adopted according to different data aggregation ratios. A useful and widely researched data aggregation scenario is that any number of packets are aggregated into a single packet. For example, calculating the average temperature or maximum temperature in the monitoring area. In this case, multiple data packets are aggregated into one data packet. This type of data aggregation problem is often abstracted as a convergecast problem [42,43]. The key character the process of collecting data is that each node only receives data in the data collection phase, and only aggregates data packets into one data packet which is sent in the data transmission phase. In the data transmission phase, regardless of the number of data packets transmitted to the nodes, no data are received by the nodes. Several convergecast algorithms [42,43] have been proposed for wireless sensor networks. Most studies divide this issue into two parts. The first part is a logical tree construction, followed by the scheduling of transmissions along the constructed tree.

Convergecast is a special case of data aggregation. The general case of data aggregation is that sensors aggregate data using a certain percentage. In the data transmission process from the source to the sink node, with continuous data aggregation, the data volume of packets increases [3,7,29,36,38]. To reduce the number of data packets, it is necessary to aggregate as many packets as possible. Villas et al. [38] proposed a data aggregation method named DRINA which improves the probability of packets routing along the same path. This method improves the probability of encountering data packets, and data are aggregated more effectively; therefore, the data size decreases. The strategy is described as follows [38]: the minimum hop routing strategy is adopted, that is, each node chooses the node with the smallest hop to the sink as the next hop for data forwarding. The formation of the minimum hop to the sink is not the same as the previous strategy. When a node has data packets to send to the sink, it chooses a routing path according to a minimum hop routing strategy. Then, the number of node hops on this path is set to 0. With hop spreading, the data in the nodes in this path route to the sink along this path. As a result, the probability of packets routing along the same path increases.

The method proposed by Kim et al. [29] to improve data aggregation performance is different from the methods mentioned above. When a node receives data packets, it does not send them immediately but holds them for a period of time. In this waiting time, data packets arrives at the data packet queue for the node. The aggregation rate can be improved by the method, but this method increases the delay. The longer the waiting time is, the higher the data aggregation rate is. However, long waiting time causes an increase in the delay [44]. This method is only tested for networks with a star topology, and simulated tests corroborate the theoretical claims. The application scope is confined to star networks. Therefore, we explore an optimized data aggregation method [45,46] that adapts to the current practical application with low energy consumption and a small delay.

### 2.2. Research on Delay Optimization

Delay in WSNs refers to the difference between the time when the sensor nodes’ data packets are generated and the time when the data packets are received by the sink. Emergencies and events in industrial production are monitored by WSNs. There are exacting requirements for the delay in WSNs, especially the WSNs for real-time monitoring. A data transmission delay could lead to catastrophic consequences. In the battlefield, fire monitoring, and factory automation control, the monitoring information is guaranteed to transfer quickly to the control center if an emergency occurs; otherwise, significant personal and property losses can be caused in the event of an enemy invasion, fire, or factory crash. Therefore, various methods to reduce delay are proposed.

#### 2.2.1. Methods for Reducing Delay in a Wireless Unreliable Transmission Environment

Due to manufacturing costs and energy limits, the structure of sensor nodes is simple, and communication power is limited. Therefore, the wireless communication between nodes is often not reliable, sometimes as low as 60–90%. In this circumstance, methods with high data transmission reliability are used. These methods are divided into many categories:

(a) Retransmission mechanism. With the retransmission mechanism, the sender retransmits the data packets which are confirmed as lost [44]. The reliability of data transmission is improved by sending data many times. The first implementation of the retransmission mechanism is the send-wait retransmission mechanism. In this mechanism, the sender waits for the receiver to return an ACK that represents the packet sent by the sender has been received. If the sender successfully receives the ACK, this means that the receiver has received the data packet and the sender is able to send the next data packet. If the sender sends a packet and does not receive an ACK after a period of time, the packet is considered lost and is retransmitted. This phenomenon is called timeout retransmission. The process of sending a packet continues until the sender receives the receiver’s ACK, or the number of retransmissions is greater than the predetermined threshold. After this step, the next packet is sent. The retransmission mechanism has an impact on the delay during the process of data transmission. It takes a long time before retransmission occurs, so delay increases due to repeated retransmissions. Networks with low transmission reliability experience great delays since a variety of retransmissions occur. To improve the defect of delay in the retransmission mechanism, several improved retransmission mechanisms are proposed. For example, the sender sends n packets at one time, and the receiver returns the sequence number of the received packets to the sender. Then, the sender retransmits the packets with sequence numbers that are not returned. We present a retransmission mechanism in [44] in which delay is reduced. If the ACK is lost, superfluous data packets are transmitted by the sender. Compared to the common data packet, the ACK load is small. We propose that the receiver returns multiple same ACKs when it receives a data packet. Thus, the probability that the sender receives the ACK increases, the number of unnecessary retransmissions and the energy consumption decrease. Additionally, the total delay decreases since the number of retransmissions decreases.

(b) Data encoding mechanism. In the retransmission mechanism, the delay is long because of repeated retransmission. The main character of the data encoding mechanism is redundancy encoding [47]. Data can be recovered using redundant code even if the data are partially lost or incorrect. The drawback to this method is that the data packets are longer than the common data packets. The higher the redundancy, the higher the probability that the receiver recovers the data packet. However, the more redundant data the node sends, the more energy that is consumed [47,48,49].

#### 2.2.2. The Delay in Duty Cycle Based Wireless Sensor Network

In many studies, nodes are always in a awake state, but sensor nodea are small, low cost, and battery powered. Their battery capacity is limited. The energy consumption of nodes in a wake state is 100–1000 times higher than in a sleep state. To save energy, we should try to keep nodes in a sleep state as much as possible. However, nodes stop sensing and communicating during the sleep state. One method used to save energy adopts the duty cycle mechanism [35]. Nodes periodically switch between the sleep state and the wake state. The ratio between the length of time of the wake state and the length of time of the whole cycle is called the duty cycle. The smaller the duty cycle, the more energy the nodes save. However, the disadvantage of this method is that delay increases. This is because the sender pauses sending data when the receiver is in a sleep state. There are several approaches to reduce delay in the duty cycle network. The basic idea of these methods is a tradeoff between network lifetime and delay. In [50], we proposed a method to reduce the delay in the dynamic duty cycle. There is residual energy in nodes which are far away from the sink in the WSNs. Increasing the duty cycle of the nodes reduces delay and does not affect the network lifetime.

#### 2.2.3. Research on Delay Optimization for Adjusting Node Transmission Power

All of the above studies are based on unchanged transmission power. The transmission power of sensor nodes is adjustable; therefore, we use this characteristic to reduce delay. Because the receiving rate of the data packets is related to the transmission power of the sender, it is a useful method for increasing the transmission power of the node. High transmission power indicates a high signal to noise ratio (SNR). Thus, the success rate of receiving data packets increases, and the number of retransmissions and delay decreases. However, the lifetime of the network is affected by the transmission power. Therefore, this relationship between transmitting the power and delay reduction [2,26,51] should be studied.

#### 2.2.4. Research on Delay in Data Aggregation

Studies on delay in data aggregation address that any number of data packets that are aggregated into one packet [42,43]. In this circumstance, only one packet is generated in a data collection cycle. A node aggregates all the data packets produced by its child node into one packet and sends it. In a cycle, nodes in the network send data in one time slot, while in other time slots, the network is monitored for receiving data. TDMA is widely used in these networks, and delay is the minimum number of slots needed in a data collection cycle. One time slot is the time it takes a node to send or receive a data packet. There are many studies in this area. For example, Huang et al. [45] proposed an algorithm with an upper bound of 23R+△-18 time slots in delay of aggregation, where R is the network radius and △ is the maximum node degree. Xu et al. [46] proposed an algorithm and proved the delay of the aggregation schedule generated by their algorithm was at most, 16R+△-14 time slots.

We also proposed a protocol named the broadcasting combined with multi-NACK/ACK (BCMN/A) protocol which combines the energy efficiency and minimizes delay under a statistically reliable constraint in [41]. Previous research is based on reliable wireless communications, but packets are often lost in wireless communication. The objective of the BCMN/A protocol is to minimize the delay in unreliable wireless communication during the data collection process. In the BCMN/A protocol, the network is optimized using the following methods. Data collection is divided into two parts, intra-cluster data collection, and inter-clusters data collection. After intra-cluster data collection, the cluster head nodes broadcast NACK to inform the nodes which fail to send data to resend the data packets. The method of sending multiple NACKs is adopted to reduce the energy consumption of the cluster head node and the total delay by decreasing the frequency of ACK transmission. In the inter-cluster data collection, multiple same ACKs are returned when a data packet is received by the receiver. Although the number of ACKs increases, the number of retransmitting data packets and the energy consumption of the cluster head nodes decrease. As a result, the lifetime of the network increases, and the total delay decreases.

In the clustering network, finding an energy-efficient policy to opt cluster heads (CHs) in the WSNs has become increasingly important. This importance is closely related to such factors as the network lifetime and efficiency. LEACH [52] is a classic cluster head selection algorithm, but it does not consider the nodes’ heterogeneities. Accordingly, SEP is proposed for clustered heterogeneous wireless sensor networks. Based on SEP, prolong-SEP [53] etc. are designed to increase the lifetime. In the field of ad hoc networks, dynamic Doppler velocity clustering (DDVC) [54] and the hierarchical clustering algorithm (HCAL) [55] are proposed to improve clustering stability and performance of the network.

#### 2.2.5. Other Research Related to Delay

Some strategies, though not designed to reduce delay, also help reduce delay. For example, the multipath routing strategy [7,32]. This routing strategy was proposed for network attacks. To prevent the attacker from dropping packets, the data is transmitted on multiple routing paths from the source to the sink. The time difference from when the first data packet reaches the sink is the delay of data transmission. This research is related to the research in this paper. The current WSNs have been rapidly developed in combination with edge networks [56,57], IoT [58] and cloud networks [59,60,61,62,63], which are the main networks for data collection [64].

## 3. System Model and Problem Statement

### 3.1. System Model

The application scope of the AAR scheme is the planar WSNs. Similar to most research, the planar WSNs can be abstracted as tree networks, in which the sink is the root of the tree network [39,40], but it’s not a node in the network. All of the data packets produced by these sensors are aggregated and transmitted to the sink. A model contains m layers, and each layer has $n_{i}$ nodes. This is shown below in Equation (1):

(1) $$\Sigma =\left[\begin{array}{cccc} \sigma_{2}^{1} & \sigma_{2}^{2} & \cdots & \sigma_{2}^{n_{2}}\\ \sigma_{3}^{1} & \sigma_{3}^{2} & \cdots & \sigma_{3}^{n_{3}}\\ \vdots & \vdots & \ddots & \vdots \\ \sigma_{m}^{1} & \sigma_{m}^{2} & \cdots & \sigma_{m}^{n_{m}} \end{array}\right]$$

**Figure 1:**
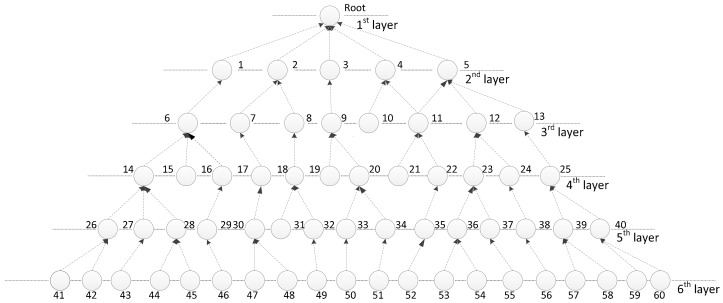
Instance of the system model.

Each node has its fixed level and a relative position, and there is only one default parent node. This tree-shaped wireless network is illustrated in Figure 1. As shown in Figure 1, the network has six layers and 60 nodes. Each node has a default parent node. For example, node 34 is the parent node of node 51.

The activities of the nodes in the network are periodic. In the data collection process U, the cycle 𝓊 is divided into the packet generation period ${𝓋}_{1}$ and the aggregation and transmission period ${𝓋}_{2}$:

(2) $$U=\left\{{𝓊}_{1},{𝓊}_{2},\ldots ,{𝓊}_{n}\right\},𝓊=\left\{{𝓋}_{1},{𝓋}_{2}\right\}$$

Each node in the data packet generation period ${𝓋}_{1}$ receives the data aggregation queues sent by their child nodes, and at most, one data packet is produced by the nodes. Each node in the aggregation and transmission period determines whether to aggregate and transmit. The transmission of the aggregation queue is performed on two adjacent layers. A node that sends the aggregation queue is $\sigma_{i}^{j}$, and a node which receives the queue is considered to $\sigma_{i-1}^{k}$ during an aggregation and transmission period. We set a unit time as a cycle 𝓊. The cycle of all nodes is synchronous. In this system, the nodes in the network are homogenous.

*Definition 1 (probability of generating a packet)*

The attribute of applications is modeled using statistical analysis [29]. For example, a wearable device generates packets for a period of 30 percent of the entire cycle, and no packet is generated during the rest period. Thus, the traffic is modeled as a successive Bernoulli trial based on a discrete time index t with a certain probability $P_{\alpha }$ [65]. Probability of generating a packet is defined as the probability that a node generates a packet in a cycle. In a packet generation period, the packets of all nodes in the model are generated as shown in Equation (3):

(3) $$C=\left[\begin{array}{cccc} c_{2}^{1} & c_{2}^{2} & \cdots & c_{2}^{n_{1}}\\ c_{3}^{1} & c_{3}^{2} & \cdots & c_{3}^{n_{2}}\\ \vdots & \vdots & \ddots & \vdots \\ c_{m}^{1} & c_{m}^{2} & \cdots & c_{m}^{n_{m}} \end{array}\right]\;c\in \left\{0,1\right\}$$

*Definition 2 (data aggregation ratio)*

**Figure 2:**
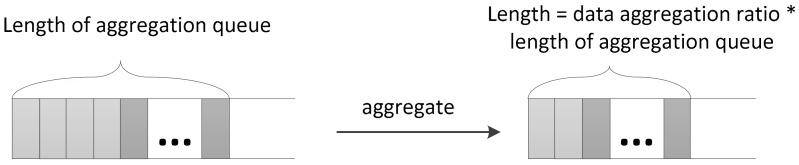
Length of the aggregation queue is reduced by aggregation.

After data aggregation, the redundant data is removed, and the packet queue length $l_{i}^{j}$ sent by the nodes in the lower layer is smaller than the original queue length. The ratio of the length of the queue after data aggregation to the length before data aggregation is defined as the data aggregation ratio λ (see from Figure 2).

The packets queue transmitted by the nodes is denoted as Equation (4):

(4) $$Q=\left[\begin{array}{cccc} q_{2}^{1} & q_{2}^{2} & \cdots & q_{2}^{n_{2}}\\ q_{3}^{1} & q_{3}^{2} & \cdots & q_{3}^{n_{3}}\\ \vdots & \vdots & \ddots & \vdots \\ q_{m}^{1} & q_{m}^{2} & \cdots & q_{m}^{n_{m}} \end{array}\right]\;q=\{\begin{array}{c} \lambda \cdot l(\sigma_{i}^{j})\\ 0 \end{array}$$

*Definition 3 (packet aggregation threshold)*

**Figure 3:**
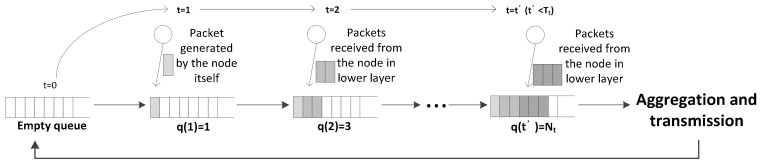
Packet aggregation occurs since the number of packets is equal to or greater than $N_{t}$.

The nodes in the model store the incoming data packets in the aggregation queue. If the number of queued packets is the same as or greater than the predetermined packet aggregation threshold, the queued packets are transmitted as an aggregated packet queue in the aggregation and transmission period (see from Figure 3). The aggregation threshold refers to the maximum number of packets aggregated into a single frame. We denote this predetermined packet aggregation threshold as $N_{t}$.

*Definition 4. (Value of the packet aggregation timer)*

**Figure 4:**
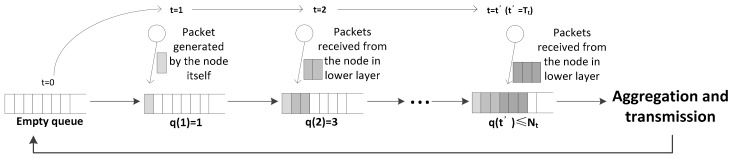
Packet aggregation occurs since the value of the aggregation timer is equal to $T_{t}$.

Because of the characteristics of the wireless network, variable waiting (or queuing) delay occurs during the aggregation process. If the time taken for the number of queued packets is the same as, or greater than packet aggregation the threshold is excessively long, the aggregation delay at the aggregator significantly increases, and the QoS deteriorates [29]. To prevent this excessive delay, the node transmits an aggregated frame when the aggregation timer expires, although the number of packets in a queue is less than the packet aggregation threshold (see from Figure 4). The aggregation timer refers to the maximum allowable time to wait for packets before transmission. We denote the value of the packet aggregation timer as $T_{t}$.

### 3.2. Problem Statement

(1) Maximize the efficiency of data packet aggregation.

In the network, increasing the aggregation efficiency is an effective way to improve the QoS and reduce the delay and the energy consumption. ℘ is considered the upper limit of the number of aggregations, and the number of aggregations performed in a node in the network is γ. The formula to maximize the data packet aggregation efficiency Ξ can be expressed by the following Equation (5):

(5) $$Max\left(\Xi \right)=\min\frac{℘}{\sum_{i=2}^{m}\sum_{j=2}^{n_{i}}\gamma_{i}^{j}}$$

(2) Minimize the delay during the data collection process.

This optimization method aims to reduce the total network transmission delay D. The total delay in the AAR scheme is the sum of delay $D_{i}^{j}$, which is shown as the following Equation (6):

(6) $$Min\left(D\right)=Min\left(\sum_{i=2}^{m}\sum_{j=2}^{n_{i}}D_{i}^{j}\right)$$

In the AAR scheme, the delay of each node is reduced. The delay in the common scheme is considered as $D_{i}^{j}$. It can be described by Equation (7):

(7) $$D_{i}^{j}\leq D_{i}^{j}|\forall i\in \left[2,m\right],j\in \left[2,n_{i}\right]$$

Due to the requirement of low power consumption of the wireless network, the number of data aggregations cannot be increased excessively while optimizing the delay.

(3) Maximize network lifetime

Network lifetime is defined as the death time of the first node in the network [32,35,36]. Considering that the average energy consumption of the *i*-th node in the network is ${Ϣ}_{i}$, its initial energy is $E_{ini}^{i}$, and there are N nodes in the network. To maximize the lifetime of the network, the network lifetime of the first node to die in the network should be maximized. Therefore, Equation (8) can be obtained:

(8) $$\max\left(ℒ\right)=Max\left[\underset{1\leq i\leq N}{\min}\left(E_{ini}^{i}/{Ϣ}_{i}\right)\right]$$

In summary, the objectives of this research are as follows in Equation (9):

(9) $$\{\begin{array}{c} \mathrm{Max}\left(\Xi \right)=\min\frac{℘}{\sum_{i=2}^{m}\sum_{j=2}^{n_{i}}\gamma_{i}^{j}}\\ \mathrm{Min}\left(D\right)=\mathrm{Min}\left(\sum_{i=2}^{m}\sum_{j=2}^{n_{i}}D_{i}^{j}\right)\\ \max\left(ℒ\right)=\mathrm{Max}\left[\underset{1\leq i\leq N}{\min}\left(E_{\mathrm{ini}}^{i}/{Ϣ}_{i}\right)\right] \end{array}$$

## 4. Optimization Mechanism Design

To clearly state the parameters of this paper, the parameters introduced in this paper can be found in Table 1.

We propose the AAR scheme to reduce the total delay in wireless networks. A detailed description of the AAR scheme is given below. In the process of data transmission in two adjacent layers, the scheme first selects the nodes with a long data queue. Next, these nodes’ priorities are set by the length of the data queue. The aggregation queues are assigned to these nodes by the priority. In the process of queue assignment, the AAR scheme ensures that the nodes receive sufficient data packets and perform data aggregation. As a result, the frequency of aggregation increases and the delay is significantly reduced.

In the common scheme, each node only receives data packets from the fixed child node and sends the aggregation queue to the fixed parent node. It is possible that the waiting queue in the node is long but it cannot receive sufficient data packets to perform aggregation, thus delay increases excessively. The AAR scheme solves this problem.

To achieve the AAR scheme, we propose a node assignment algorithm (NAAL), which is based on the greedy strategy. We describe and explain this algorithm in Section 4.1, then analyze the complexity of NAAL in Section 4.2. Finally, we provide an instance to demonstrate this algorithm.

### 4.1. Description and Remarks about NAAL

The aim of NAAL is to assign the aggregation data queues to the nodes with sufficiently long data waiting queues to make them aggregate data. We set a parameter Ω. If the ratio of the length of the waiting queue to the aggregation threshold $N_{t}$ is equal to or greater than Ω and less than 1, the node receives the queue. The proposed algorithm is given below in Algorithm 1.

**Algorithm 1** Node assignment algorithm1:**For** each $\sigma_{i}^{j}$| $j\;from\;1\;to\;n_{i}$//collect all the nodes whose aggregation queue is ready to aggregate.2: **If**
$l(\sigma_{i}^{j})\geq Nt_{i}^{j}$ or $t(\sigma_{i}^{j})\geq Tt_{i}^{j}$
**then**3:  $\sigma_{i}^{j}\in \Sigma_{i}$4: **End if**5:
**End for**
6:**If**$\Sigma_{i}=\emptyset$**then**//if $\Sigma_{i}=\emptyset$, no node in $L_{i}$ send the aggregation queue.7: **Return**8:
**End if**
9:**For** each $\sigma_{i-1}^{k}|k\;from\;1\;to\;n_{i-1}$//collect all the nodes whose $\Omega \leq \omega_{i-1}^{k}<1$.10: **If**
$\Omega \leq \omega_{i-1}^{k}<1$
**then**11:  $\sigma_{i-1}^{k}\in \Sigma_{i-1}$12: **End if**13:
**End for**
14:**For** each $\sigma_{i-1}^{k^{\prime }}|$ from $\sigma_{i-1}^{k^{\prime }}$ with max $\omega_{i-1}^{k^{\prime }}$ to $\sigma_{i-1}^{k^{\prime }}$ with min $\omega_{i-1}^{k^{\prime }}$ in $\Sigma_{i-1}$//assign $\sigma_{i}^{j^{\prime }}$ to $\sigma_{i-1}^{k^{\prime }}$ with the priority of large $\omega_{i-1}^{k^{\prime }}$ to small.15: **While**
$l(\sigma_{i-1}^{k^{\prime }})<N_{t}\;\mathrm{and}\;\Sigma_{i}\neq \emptyset$
**then** //assign $\sigma_{i-1}^{k^{\prime }}$ with sufficient aggregation queues to make the node aggregate.16:  $l(\sigma_{i-1}^{k^{\prime }})=l(\sigma_{i-1}^{k^{\prime }})+l(\sigma_{i}^{j^{\prime }})*\lambda$17:  $\sigma_{i}^{j^{\prime }}\notin \Sigma_{i}$18: **End while**19: $\sigma_{i-1}^{k^{\prime }}\notin \Sigma_{i-1}$20:
**End for**
21:**If**$\Sigma_{i}\neq \emptyset$**then**//the remaining queues of nodes in $\Sigma_{i}$ are transmitted to $\tau (\sigma_{i}^{j})$.22: **While**
$\Sigma_{i}\neq \emptyset$
**then**23:  $l(\tau (\sigma_{i-1}^{j^{\prime \prime }}))=l\left(\tau (\sigma_{i-1}^{j^{\prime \prime }}\right))+l(\sigma_{i}^{j^{\prime \prime }})*\lambda$24:  $\sigma_{i}^{j^{\prime \prime }}\notin \Sigma_{i}$25: **End while**26:
**End if**


The explanatory remarks about Algorithm 1 are shown below:

*Line 1–5*: When traversing a layer $L_{i}$, all the nodes in this layer that meet the aggregation criteria are moved to $\Sigma_{i}$.

*Line 6–8*: If $\Sigma_{i}$ is empty, the algorithm would return.

*Line 11–13*: select the nodes in $L_{i-1}$ whose $\omega_{i-1}^{k}$ is greater than or equal to Ω and less than 1 to $\Sigma_{i-1}$.

*Line 14–20*: Nodes in $\Sigma_{i-1}$ are prioritized with ω from large to small. Next, assign the aggregation queue of node in $\Sigma_{i}$ to $\sigma_{i-1}^{k^{\prime }}$ by priority and remove the node from $\Sigma_{i}$ until all the aggregation queues provided by nodes in $\Sigma_{i}$ makes $l(\sigma_{i-1}^{k^{\prime }})\geq N_{t}$, then remove $\sigma_{i-1}^{k^{\prime }}$ from $\Sigma_{i-1}$.

*Line 21–26*: If there are nodes remaining in $\Sigma_{i-1}$ which have no queue to receive, the algorithm ends. If $\Sigma_{i}$ is not empty, transmit each aggregation queue to its parent node by default $\tau \left(\sigma_{i}^{j^{\prime \prime }}\right)$.

### 4.2. NAAL Complexity Analysis

We define the number of nodes in the network as N, the number of nodes in layer i is $N_{i}$. The number of layers in the network is m. First, the nodes in layer i which perform aggregation are moved to $\Sigma_{i}$. The overhead of this step is $O\left(N_{i}\right)$. Then, we move the nodes with a long data queue in layer i−1 to $\Sigma_{i-1}$. The time complexity is $O\left(N_{i-1}\right)$. After that, the nodes in $\Sigma_{i-1}$ are sorted by the length of the data queue. The time complexity is $O\left(\log N_{i-1}\right)$. Finally, assign the data queues to the nodes in $\Sigma_{i-1}$ and handle with the remaining nodes in $\Sigma_{i-1}$ or $\Sigma_{i}$. It performs the nodes traversal in $\Sigma_{i}$ and $\Sigma_{i-1}$. The time complexity is $O\left(N_{i}+N_{i-1}\right)$. Therefore, the time complexity of assigning the data queues in the whole network one time using the proposed algorithm is shown as Equation (10):

(10) $$O\left(2\overset{i=m-1}{\underset{i=2}{\sum }}N_{i}+2\overset{j=m}{\underset{j=3}{\sum }}N_{j}+log\overset{i=m-1}{\underset{i=2}{\sum }}N_{i}\right)=O\left(N\right)$$

### 4.3. Illustration of NAAL

**Figure 5:**
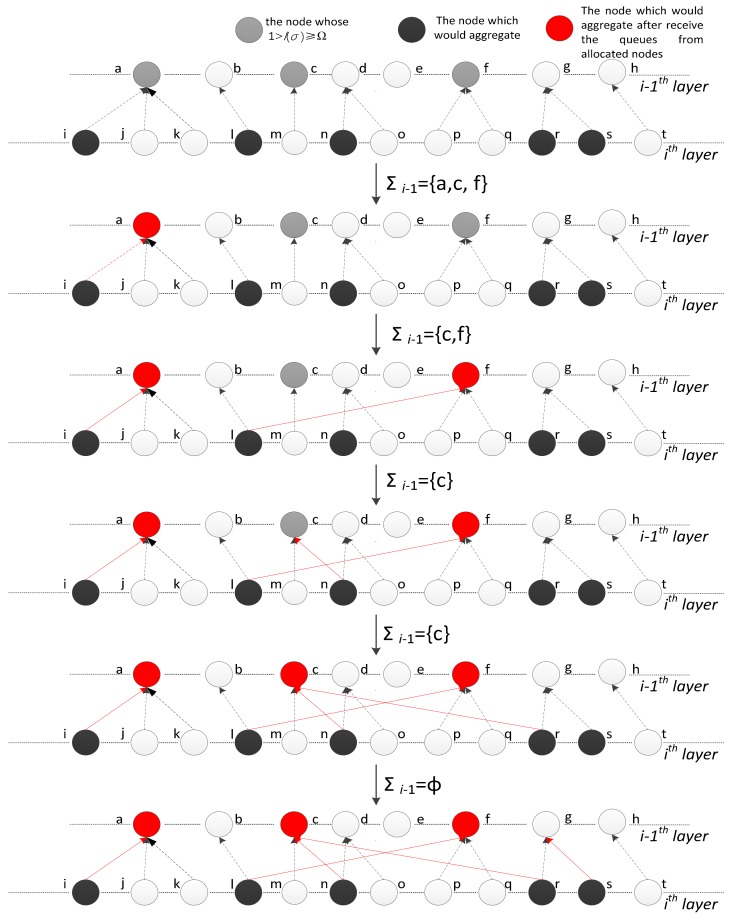
Demonstration diagram of the algorithm.

The detailed process of the algorithm is clarified in this sub-section. As revealed in Figure 5, the nodes of the two layers are described, $\Sigma_{i-1}$ is {a, c, f}, and $\Omega \leq l(\sigma_{i-1}^{c})\leq l(\sigma_{i-1}^{f})\leq l(\sigma_{i-1}^{a})\leq 1$. Node i is assigned to node a and then i is removed from $\Sigma_{i}$. At this time, the queue of node a can be aggregated, and a is removed from $\Sigma_{i-1}$. Next, assign node m to f and remove m from $\Sigma_{i}$. The sum of the length of the queue in f and the length of the queue received from m is greater than $N_{t}$, exclude m from $\Sigma_{i-1}$. The queue of n is assigned to c and n is excluded from $\Sigma_{i}$. However, c must obtain additional data to perform aggregation. Thus, the data queue of r is assigned to be transmitted to c. The queue meets the criteria of aggregation, exclude c from $\Sigma_{i-1}$. Now, $\Sigma_{i-1}$ is an empty set, but node s is still in $\Sigma_{i}$. Therefore, *s* would transmit the queue to its default parent node g.

## 5. Performance Analysis and Optimization

### 5.1. Methodology and Setup

In this section, we compare the AAR scheme with the common scheme (CS) regarding total delay, the number of aggregations and the lifetime. The main characteristic of CS is that each node’s aggregation queue can only be sent to the default parent node. We performed Monte Carlo simulations to validate the optimization performance of our method. First, the simulation process generates a tree network randomly. Each node has a fixed parent node, which is used to receive the queues sent by the node. Fifty thousand packets are generated in the network, and when all these packets are received by the root, it is regarded as the end of a simulation. We set a cycle as a unit time that contains one period of packet generation and one period of aggregation.

The network parameters used in our experiments are listed in Table 2. The simulation networks include different numbers of layers and nodes. m=3 indicates that the number of layers in the network is 3. To evaluate the performance of the AAR scheme in different environments, we build three scenarios.

**Figure 6:**
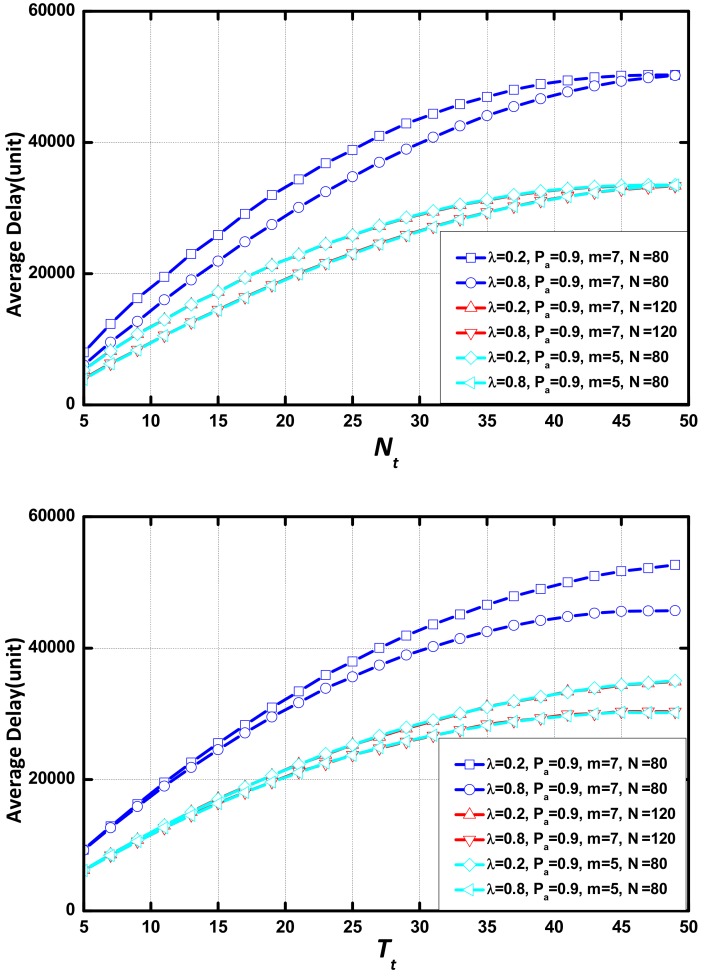
Average delay in various circumstance vs. $N_{t}$ or $T_{t}$.

The parameters which determine the scale of the network are m and N. N/m is used as the indicator that measures the structure of the tree. A large value of N/m means that this is a fat network. The reason for using this indicator is as follows: we set three kinds of network, (m, N) = {(7, 80), (7, 120), (5, 80)}. The value of N/m is 1.33, 1.5, 1.5 (the sink is in the first layer, but it is not a node in the network). As shown in Figure 6, the networks with the same N/m have the same value of average delay.

The scenarios in the simulation are: (m, N) = {(3, 20), (5, 60), (7, 80)}. The values of N/m of the three scenarios are 1.0, 1.5, 1.33 respectively. λ, $P_{\alpha }$ are considered as the environmental parameters in the simulation. The values of λ and $P_{\alpha }$ are selected randomly.

We first study the performance of the optimization in various cases in terms of delay in Section 5.2. In Section 5.3, we study the effect of Ω on the optimization performance. Finally, we compare the performance of the AAR scheme and the improved AAR scheme in Section 5.4. All simulations using the same settings are repeated twenty times to get the average values.

### 5.2. Optimization Performances on Delay

We aim to investigate the optimization effect of the AAR scheme in different circumstances. We analyze the optimization performance of the AAR scheme in different scenarios in terms of delay. The effect of environmental parameters, λ and $P_{\alpha }$, on delay and the number of aggregation is tested. Next, the optimization performance of delay and lifetime and the effect on the number of aggregation are presented. The lifetime of the networks using these two schemes are compared. In this sub-section, Ω is a constant with the value of 0.4.

#### 5.2.1. The Effect of Environmental Parameters on Delay

**Figure 7:**
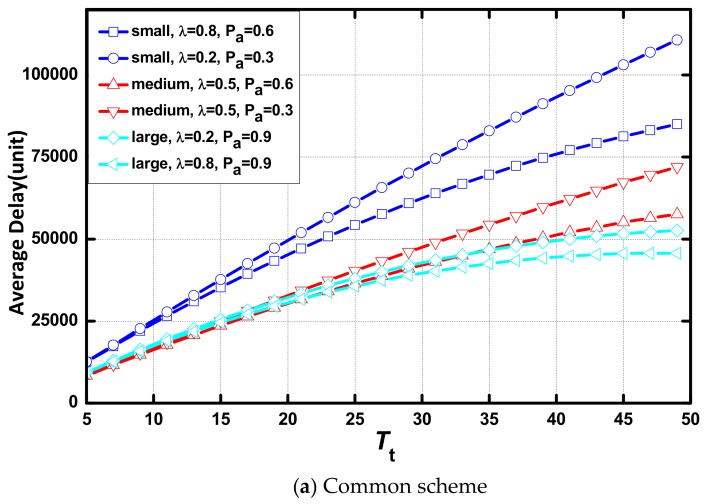

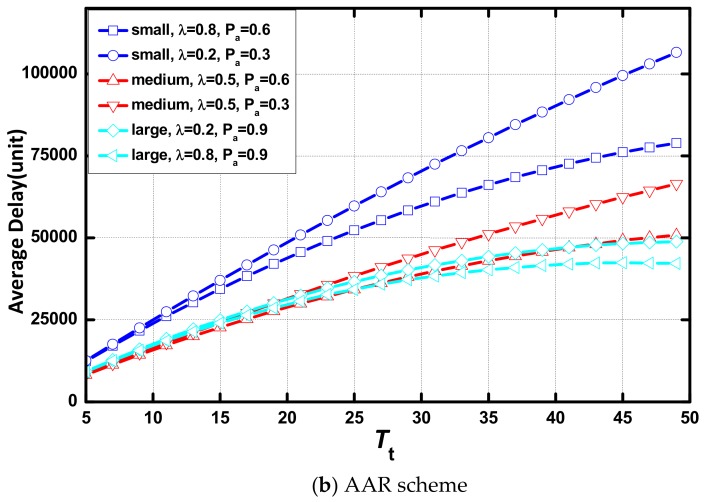
Average delay vs. $T_{t}$ in different scenarios.

As shown in Figure 7, delay increases linearly as $T_{t}$ increases. When $T_{t}$ is small, there is no difference in the delay between networks in different environments in each scenario and the difference increases with the increase of $T_{t}$. In the same scenario, the network with a large value of λ or $P_{\alpha }$ presents a small delay. The increasing rate of delay is lower, and increasing delay disappears faster when the value of λ or $P_{\alpha }$ increases. In addition, the AAR scheme causes no change to the effect of $T_{t}$ on delay.

**Figure 8:**
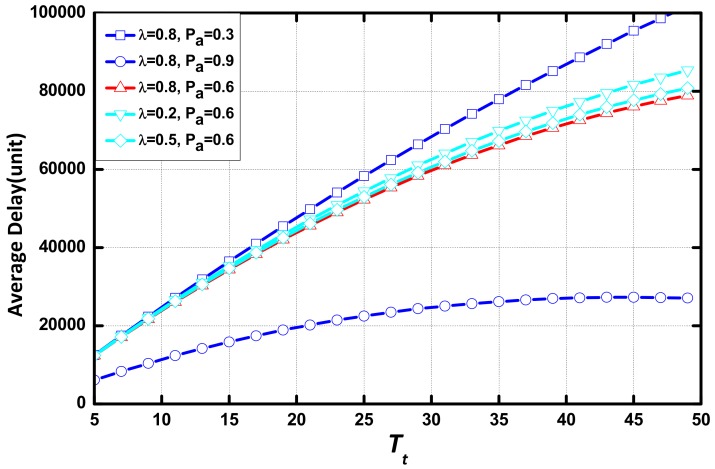
Average delay vs. $T_{t}$ with different (λ, $P_{\alpha }$).

In Figure 8, we compare the delay of the networks in various environments in the same scenario. The values of the environmental parameters is: (λ, $P_{\alpha }$) = {(0.8, 0.3), (0.8, 0.9), (0.8,0.6), (0.2, 0.6), (0.5, 0.6)}. It can be seen that $P_{\alpha }$ is the environmental parameter that determines the delay, and λ has little effect on delay. Delay is affected significantly by the change of $P_{\alpha }$ when the value of $P_{\alpha }$ is small.

).

**Figure 9:**
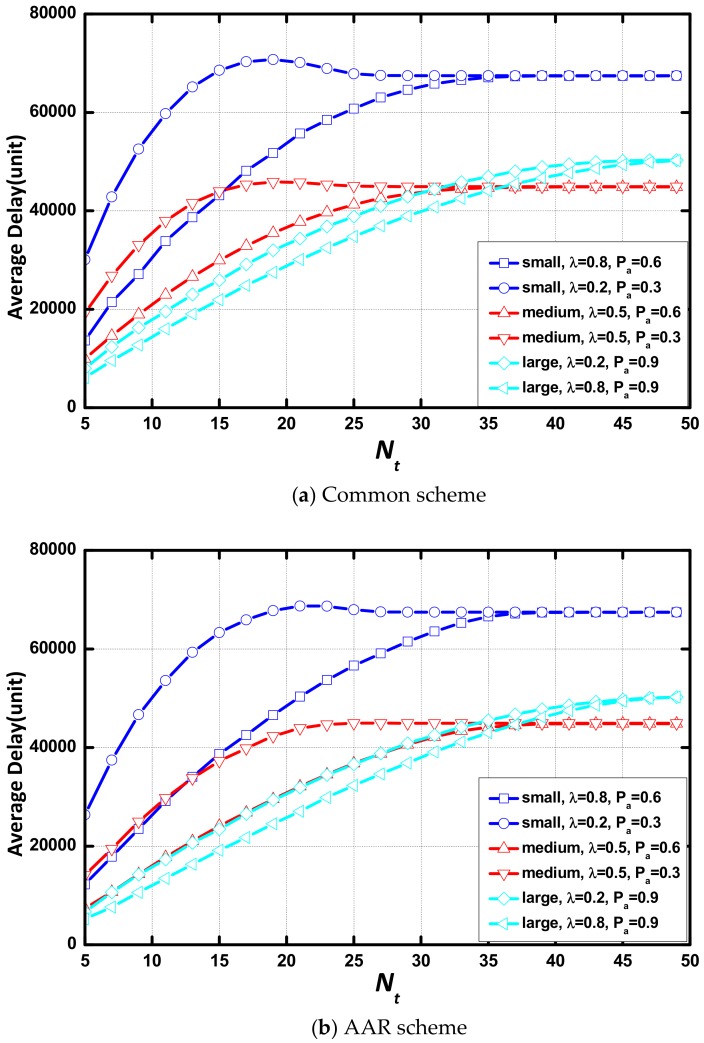
Average delay vs. $N_{t}$ in different scenarios.

Figure 9 indicate that the delay significantly increases and then remains unchanged as $T_{t}$ increases. The difference in delay between the network with different environmental parameters in each scenario increase at first and then decrease to 0. The unchanged delay points out that $N_{t}$ is not a parameter that determines delay in these cases. $T_{t}$ is the only parameter to affect delay. In this case, all aggregations occur because of the expiration of aggregation timer. The AAR scheme makes causes no change to the effect of $N_{t}$ on delay.

**Figure 10:**
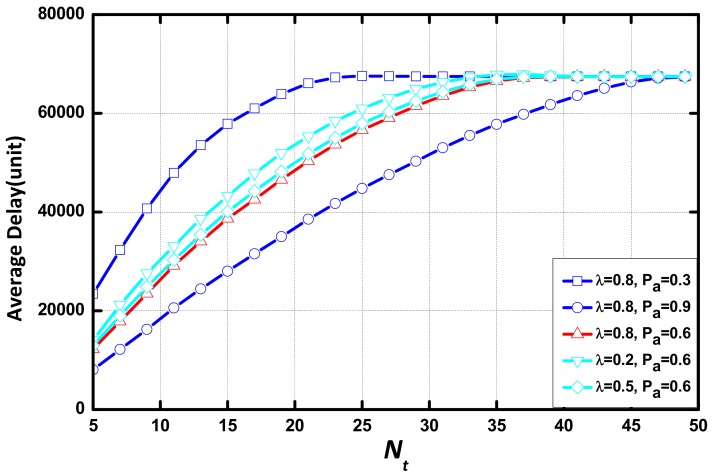
Average delay vs. $N_{t}$ with different (λ, $P_{\alpha }$) when (m,N) = (3,20).

Figure 10 compares the delay of the networks in various environments. It can be seen that $P_{\alpha }$ is the major environmental parameter. However, delay is saturated at a large aggregation threshold eventually.

#### 5.2.2. The Effect of Environmental Parameters on the Number of Aggregations

**Figure 11:**
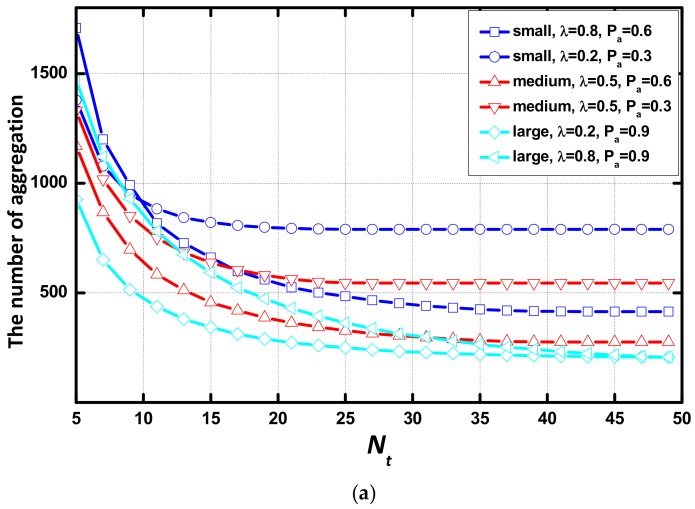

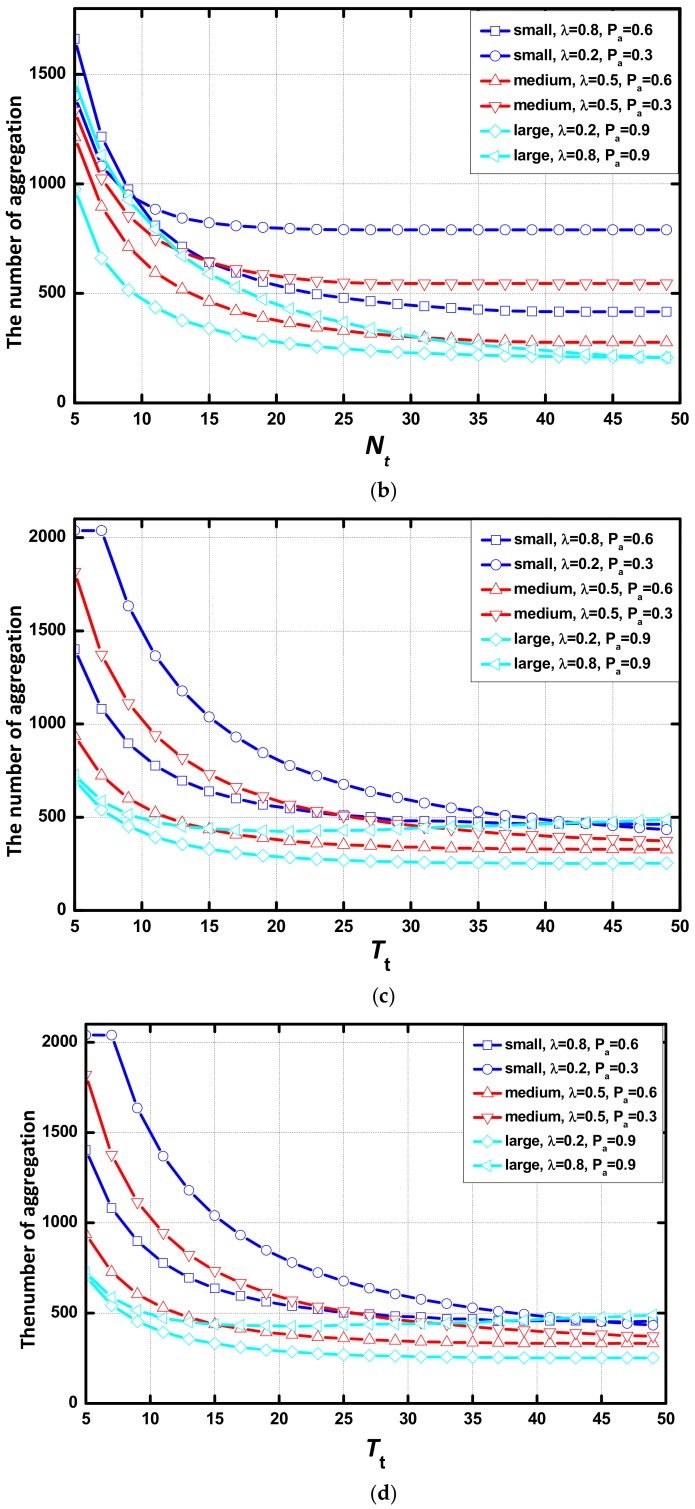
Number of aggregations vs. $N_{t}$ or $T_{t}$ in different environments (**a**) Common scheme, vs. $N_{t}$; (**b**) AAR scheme, vs. $N_{t}$; (**c**) Common scheme, vs. T_t_; (**d**) AAR scheme, vs. $T_{t}$.

In this subsection, we study the effect of environmental parameters regarding the number of aggregations. As revealed in Figure 11, the number of aggregations rapidly decreases at first but is saturated at a sufficiently large value of $N_{t}$ and $T_{t}$. By the contrast of (a), (b) and (c), (d), with the increase of $T_{t}$, the number of aggregations decreases more than with the increase of $N_{t}$. When the value of $T_{t}$ is large, the difference of the number of aggregations in these networks is smaller than when the value of $N_{t}$ is large. In each scenario, the average number of aggregation decreases with the small λ or the large $P_{\alpha }$. The number of packets transmitted in the simulations is constant. The small number of aggregations represents a high expectation of the number of data packets per aggregation. The number of aggregations decreases smoothly at a large value of λ or $P_{\alpha }$. The trend of the number of aggregations with $N_{t}$ or $T_{t}$ is opposite to that of delay, and the area that the number of aggregation doesn’t change is similar to that of delay. Additionally, the AAR scheme has no effect on the trend of the number of aggregations.

**Figure 12:**
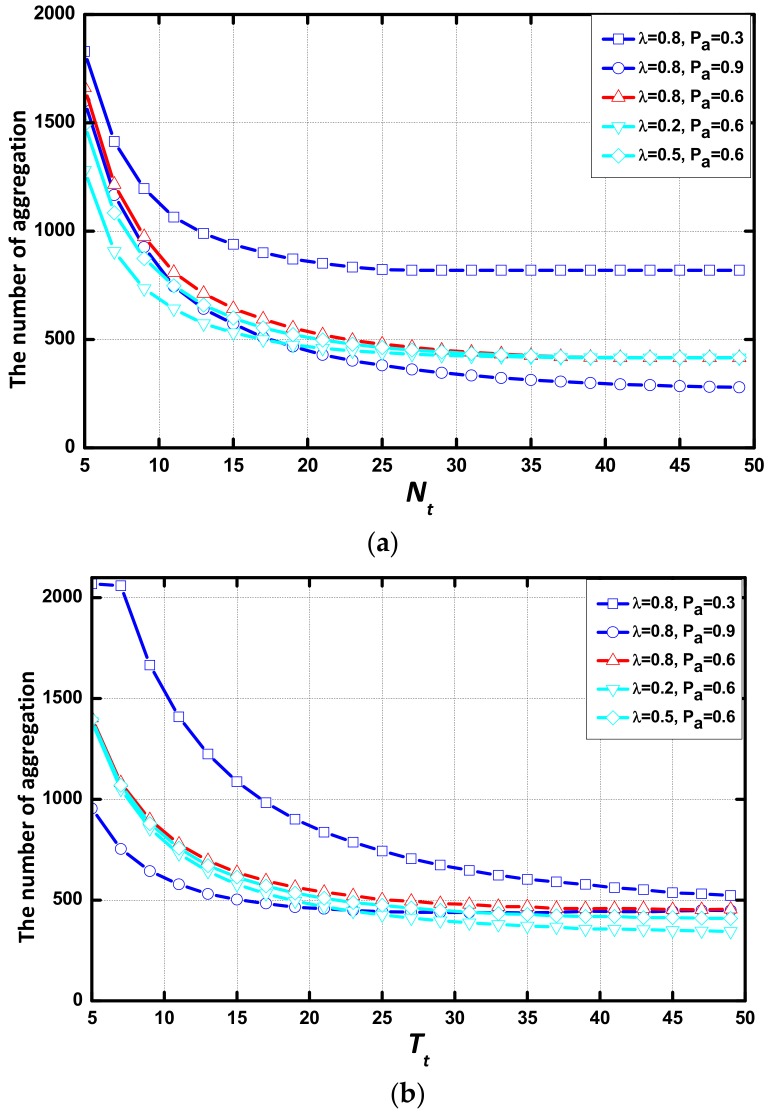
Average delay with different (λ, $P_{\alpha }$) when (m,N) = (3,20) (**a**) Average delay vs. $N_{t}$; (**b**) Average delay vs. $T_{t}$.

As seen in Figure 12, there is a gap in the number of aggregations between the networks with different $P_{\alpha }$. $P_{\alpha }$ is the major environmental parameter which determines the number of aggregations.

#### 5.2.3. The Optimization Performance of AAR Scheme

**Figure 13:**
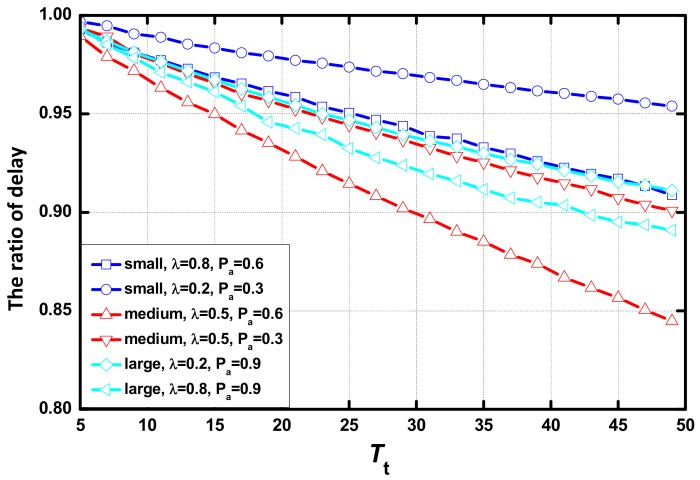
Ratio of average delay of the AAR scheme and the common scheme vs. $T_{t}$.

As seen in Figure 13, the ratio between the delay with the AAR scheme and the delay with CS linearly decreases as $T_{t}$ increases. The optimization performance of the AAR scheme increases as $T_{t}$ increases. Additionally, there is no sign that the trend would stop with the increase of $T_{t}$. In the same scenario, the network with the larger value of λ or $P_{\alpha }$ has better optimization performance.

**Figure 14:**
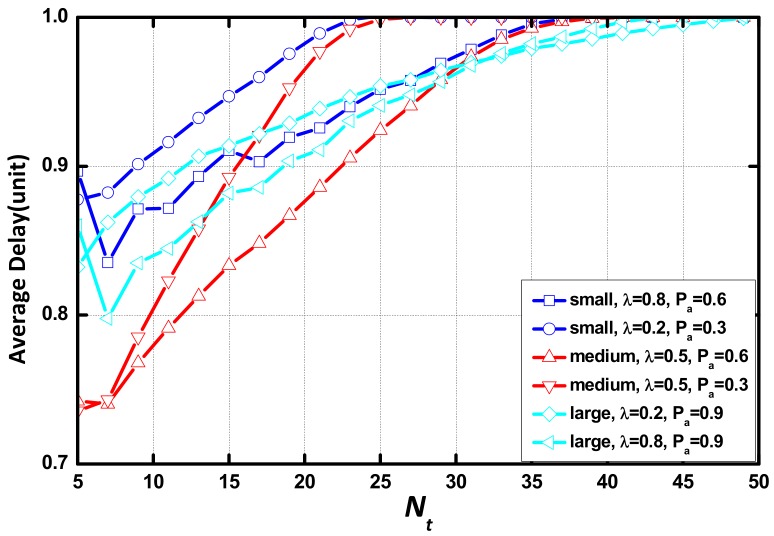
Ratio of the average delay of the AAR scheme and the common scheme vs. $N_{t}$.

Figure 14 illustrates the ratio between the delay with the AAR scheme and the delay with the CS. The horizontal bar indicates the increase of $N_{t}$. With the increase of $N_{t}$, the ratio increases rapidly and is saturated at a large value of $N_{t}$. The value of N/m is positively correlated with the mathematical expectation of the aggregation queue that each layer provide. The value of λ and $P_{\alpha }$ determine the area that has optimized performance and the value of N/m determines the performance of AAR scheme. The area where the AAR scheme has no optimization is similar to the area where delay no longer increases with the increases of $N_{t}$. In summary, the AAR scheme works well when $N_{t}$ is the parameter that determines the performance of the packet aggregation process. When $N_{t}$ and $T_{t}$ are constant, the area where the AAR scheme works is constrained by λ and $P_{\alpha }$ and the performance is positively correlated with the value of N/m.

Network lifetime is defined as the death time of the first node in the network [32,35,36]. The main energy consumption of the sensing nodes is while receiving and sending data packets, so reducing the number of aggregations can reduce energy consumption. The optimization performance in terms of lifetime is studied in this sub-section. The expectation of the maximum number of aggregations in the networks that have optimization effect are compared in Table 3. The AAR scheme decreases the maximum number of aggregations in the network, thus postponing the death of the first node in the network. Thus, the lifetime of the network increases. There are optimization effects in different scenarios, and the effect increases with the increase of λ and $P_{\alpha }$.

The average optimization performance of the AAR scheme reduces the average delay by 14.91% and the lifetime increases by 30.91% when $N_{t}$ and $T_{t}$ are the dominant parameters that determine the performance of the packet aggregation process.

#### 5.2.4. The Number of Aggregation of AAR Scheme

**Figure 15:**
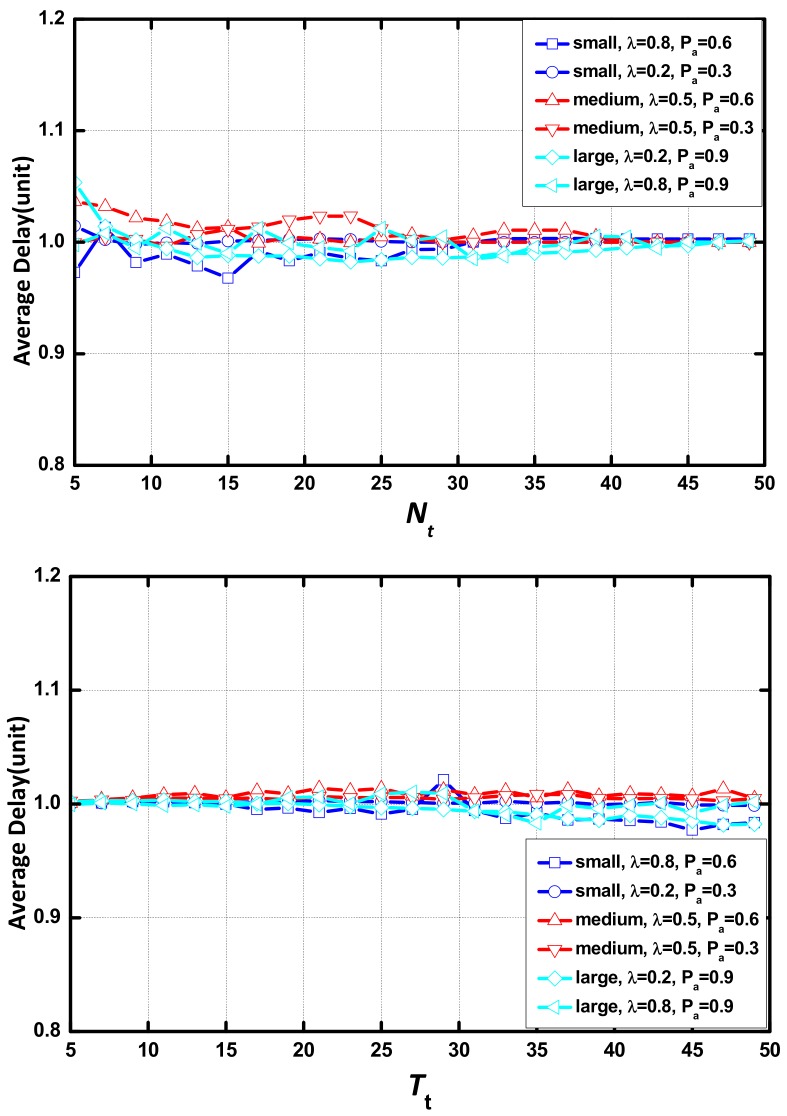
Ratio of the number of aggregations vs. $N_{t}$ or $T_{t}$.

The optimization methods should improve the QoS by consuming limited extra energy. As revealed in Figure 15, the AAR scheme has a very limited impact on the number of aggregations. The ratio of the number of aggregations is approximately 1. This means that there is no extra energy consumption regarding aggregation in the AAR scheme.

### 5.3. Performance of Optimizing at Different Node Selection Parameter

The node selection parameter Ω determines the nodes in each layer that can receive the aggregation queues. In this sub-section, the parameter is set to 0, 0.1, 0.2, …, 1.0 (in the case of Ω = 1.0, all of the aggregation queues are transmitted to the default parent node, that is, there is no optimization), we study the influence of different node selection parameters on the delay optimization.

**Figure 16:**
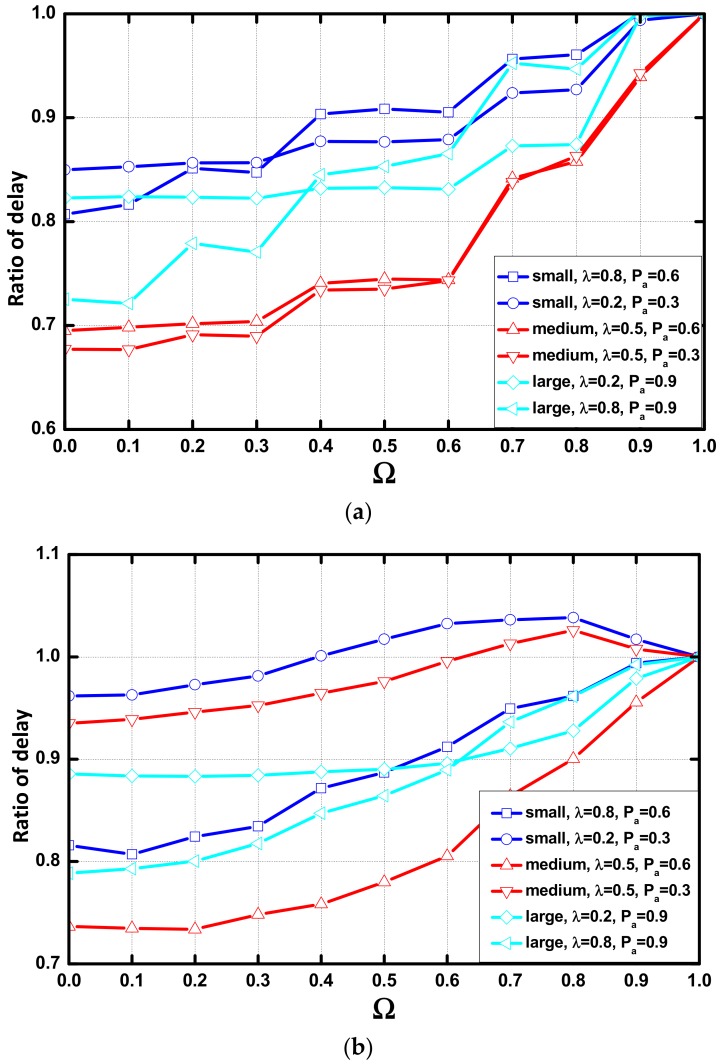

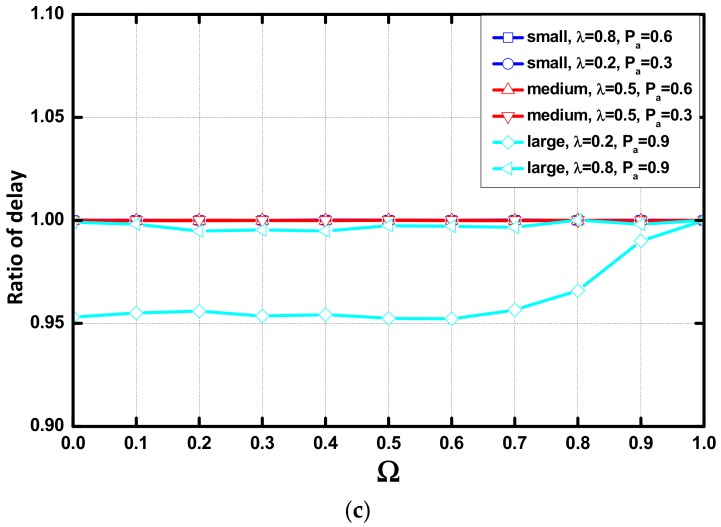
Ratio of delay vs. Ω (**a**) $N_{t}$ = 5; (**b**) $N_{t}$ = 15; (**c**) $N_{t}$ = 25.

In Figure 16a we find that the optimization performance decreases as the value of Ω increases. The performance disappears when Ω equals 1. When the value of $N_{t}$ is 15, the optimization effect on the delay also decreases when Ω increases. We notice that there is negative optimization when $P_{\alpha }$ = 0.3. In the study in Section 5.2, we determine that the large $N_{t}$ has no constraint on aggregation. $T_{t}$ is the only parameter that controls the occurrence of aggregation. Therefore, there is no optimization in these environments.

Figure 16c shows that when $N_{t}$ = 25 and $T_{t}$ = 25, there was no optimization except for the network whose λ = 0.8 and $P_{\alpha }$ = 0.9, as the area where the AAR works is large when the network has a large value of λ and $P_{\alpha }$. Therefore, performance are not affected by the value of Ω except for the network with a large value of λ and $P_{\alpha }$.

### 5.4. Performance of the AAR Scheme VS. the Improved AAR Scheme

Based on the AAR scheme, an improved AAR scheme (IMAAR) is proposed in this paper. It improves the QoS. The innovation of the IMAAR scheme is that because the data on the nodes far away from the sink use more hops to be transmitted to the sink and delay exists in every hop, delay is generated during the data collection process. Therefore, to reduce the delay, we need to reduce the delay in the process of data transmission from the nodes far from sink to the sink.

**Figure 17:**
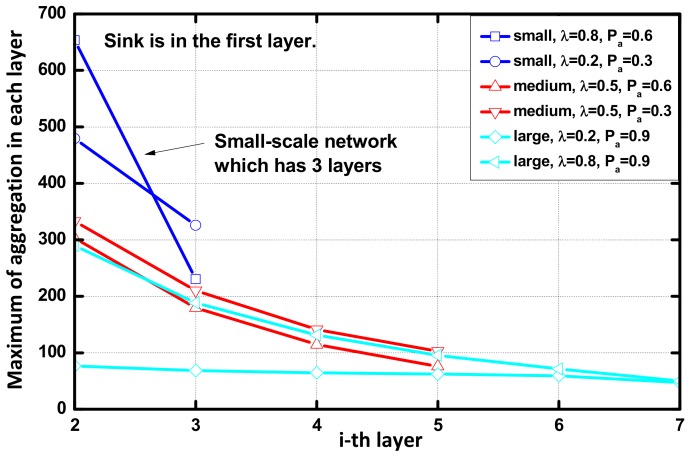
Maximum number of aggregations in each layer.

In this subsection, we propose a strategy to adjust the value of $N_{t}$ and $T_{t}$. The expectation of the maximum number of aggregations in each layer of the network using the AAR scheme are calculated. In Figure 17, we see that the number of aggregations decreases with the increase of hops to the sink. Therefore, the nodes far from the sink have remaining energy when the first node dies. In the improved AAR scheme, the nodes far from the sink are set to a smaller value of aggregation timer ($T_{t}$) and queue length threshold ($N_{t}$) to reduce delay, so the residual energy can be fully used. The decrease in the number of polymerizations is influenced by λ.

Due to the homogeneity of the nodes, the upper limit of the number of aggregations ℘ of all nodes in the network is equal. The number of aggregations performed in a node is γ. The energy efficiency Ξ can be expressed as Equation (11):

(11) $$\Xi =\frac{\sum_{i=1}^{m}\sum_{j=1}^{n_{i}}\gamma_{i}^{j}}{℘N}$$

In the AAR scheme, the value of $N_{t}$ and $T_{t}$ are set by predetermined $N_{t}$($\bar{N_{t}}$) and predetermined $T_{t}$($\bar{T_{t}}$). We design a formula to achieve the configuration of $N_{t}$ and $T_{t}$ for the node in layer i, which has shown below (the formula of $T_{t}$ is same that of $N_{t}$):

(12) $$N_{t}=\{\begin{array}{c} \bar{N_{t}}\frac{L-i{\left(\log_{2}\frac{1}{\lambda }\right)}^{3}}{L},\;\lambda >0.5\\ \bar{N_{t}}\frac{L-i}{L}\log_{2}\frac{1}{\lambda },\;\lambda \leq 0.5 \end{array}$$

We ran the simulation to evaluate the energy efficiency. The upper bound of aggregation is 500. As long as the number of aggregations of one node reaches the upper limit, the network is considered dead. The number of aggregations of each node is counted to calculate the energy efficiency. The energy efficiency of the two schemes in different network scenarios and environments is seen in Table 4. This finding shows IMAAR significantly improves the energy efficiency of the network, which is improved by an average of 76.40%.

## 6. Conclusions

In this paper, we have presented an adaptive aggregation routing (ARR) scheme with the belief that our proposed scheme is an efficient means to reduce delay and improve the lifetime for WSNs. To address different applications, this scheme can be implemented in all networks with a tree topology. In this scheme, the node assignment algorithm (NAAL) is proposed for dynamically assigning the aggregation queues in the lower layer to the nodes in the upper layer. According to the state of the data queue in the nodes, this algorithm dynamically selects nodes that receive the aggregation queues in this cycle, while other nodes in the same layer can sleep to save energy. First, the nodes with a long data packet waiting queue are selected. Next, the priority is set according to the length of the queue in these nodes. Finally, the aggregation queues are assigned to these nodes by priority. In this process, the node is guaranteed to aggregate by receiving sufficient data. This increases the aggregation frequency and decreases the delay. Considering the aggregation threshold, the aggregation timer, the data aggregation ratio and probability that produce a data packet each cycle as the dominant parameters that determine the performance of the data aggregation and transmission process, we conducted simulation experiments in different cases. The performance of the proposed network scheme is compared with the common scheme that the aggregation queues only transmit to the parent node by default. The results are consistent with our expectations. The proposed network structure is shown to be more efficient in terms of delay and lifetime during the data collection process, and this scheme does not cause extra energy consumption in aggregation. The AAR scheme works well when $N_{t}$ has a strong constraint on delay, or the value of λ or $P_{\alpha }$ is large, especially the value of $P_{\alpha }$. When $T_{t}$ is the only parameter that operates the occurrence of aggregation, the AAR scheme does not work. In the network in which the AAR scheme has optimization effect, the delay is reduced by 14.91% and the lifetime is improved by 30.91%. The node selection parameter is designed to select the nodes with a long data queue. Through the simulations, we find that the small value of the parameter performs well regarding optimization, the optimum value of the parameter is 0. Based on the AAR scheme, an improved AAR scheme (IMAAR) is proposed in this paper. The IMAAR performs well regarding energy efficiency. Thus, the residual energy is fully used. In the experiments, IMAAR improves the energy efficiency by 76.40%. To the best of our knowledge, there is no optimization method based on the state of the data queue in the node. There is room for development for optimization based on a given state of the node. We expect that this study will contribute to the development of new optimization methods.

## Figures and Tables

**Table 1:** Parameter description.

Parameter	State
$L_{i}$	All the nodes in layer i of the wireless network.
$\Sigma_{i}$	The set which contains the nodes which participate in the transmission in layer i.
$\sigma_{i}^{j}$	A node at $L_{i}$ and relative position j.
N	The number of the nodes in the network.
m	The number of the layer in the network.
$n_{i}$	The number of the nodes at $L_{i}$.
$N_{t}$	Packet aggregation threshold.
$T_{t}$	Value of the packet aggregation timer.
$\tau (\sigma_{i}^{j})$	The parent node of $\sigma_{i}^{j}$ by default.
$l(\sigma_{i}^{j})$	Current length of data packets queue of $\sigma_{i}^{j}$.
$t(\sigma_{i}^{j})$	Current value of aggregation timer of $\sigma_{i}^{j}$.
$\omega_{i}^{j}$	The ratio of the length of the current queue to $N_{t}$ in $\sigma_{i}^{j}$.
Ω	The parameter at which a node receives the aggregate data queue from the lower layer.
λ	Data aggregation ratio.
$P_{\alpha }$	The probability that a sensor generates sensing data during a packet generation period.

**Table 2:** Simulation parameters.

Parameter	Value
m	{3, 5, 7}
N	{20, 60, 80}
λ	{0.2, 0.5, 0.8}
$P_{\alpha }$	{0.3, 0.6, 0.9}
$N_{t}$	5–50 (packet)
$T_{t}$	5–50 (unit)
Ω	{0.1, 0.2, 0.3, 0.4, 0.5, 0.6, 0.7, 0.8, 0.9, 1.0}

**Table 3:** Maximum of the aggregation in two schemes.

Scenario	Maximum of the Aggregation in CS	Maximum of the Aggregation in AAR	Ratio (%)
Small, (0.2,0.3)	590.58	479.18	81.13
Small, (0.8,0.6)	891.96	653.41	73.26
Medium, (0.5,0.3)	410.97	332.92	81.01
Medium, (0.5,0.6)	390.90	302.95	77.50
Large, (0.2,0.9)	94.24	76.84	81.54
Large, (0.8,0.9)	449.90	289.80	66.41

**Table 4:** Energy efficiency of two schemes.

Scenario	Energy Efficiency of AAR (%)	Energy Efficiency of IMAAR (%)
Small, (0.2,0.3)	76.06	90.80
Small, (0.8,0.6)	48.94	82.53
Medium, (0.5,0.3)	35.85	67.55
Medium, (0.5,0.6)	35.71	68.88
Large, (0.2,0.9)	73.88	79.14
Large, (0.8,0.9)	25.98	73.25
